# Insights into the genetic basis of retinal detachment

**DOI:** 10.1093/hmg/ddz294

**Published:** 2019-12-09

**Authors:** Thibaud S Boutin, David G Charteris, Aman Chandra, Susan Campbell, Caroline Hayward, Archie Campbell, UK Biobank Eye & Vision Consortium, Priyanka Nandakumar, David Hinds, 23andMe Research Team, Michelle Agee, Babak Alipanahi, Adam Auton, Robert K Bell, Katarzyna Bryc, Sarah L Elson, Pierre Fontanillas, Nicholas A Furlotte, Barry Hicks, Karen E Huber, Ethan M Jewett, Yunxuan Jiang, Aaron Kleinman, Keng-Han Lin, Nadia K Litterman, Matthew H McIntyre, Kimberly F McManus, Joanna L Mountain, Elizabeth S Noblin, Carrie A M Northover, Steven J Pitts, G David Poznik, J Fah Sathirapongsasuti, Janie F Shelton, Suyash Shringarpure, Chao Tian, Joyce Y Tung, Vladimir Vacic, Xin Wang, Catherine H Wilson, Danny Mitry, Veronique Vitart

**Affiliations:** 1 MRC Human Genetics Unit, MRC Institute of Genetics and Molecular Medicine, University of Edinburgh, EH4 2XU Edinburgh, UK; 2 Moorfields Eye Hospital, EC1V 2PD London, UK; 3 Department of Ophthalmology, Southend University Hospital, Essex SS0 0RY, UK; 4 Vision & Eye Research Unit, Anglia Ruskin University, Essex CM1 1SQ, UK; 5 Generation Scotland, Centre for Genomic and Experimental Medicine, University of Edinburgh, Institute of Genetics and Molecular Medicine, EH4 2XU Edinburgh, UK; 6 Full list of members and affiliations is provided in S3 Text; 7 23andMe, Inc. Mountain View, Sunnyvale, CA 94041, USA; 8 Department of Ophthalmology, Royal Free NHS Foundation Trust, NW3 2QG London, UK

## Abstract

Retinal detachment (RD) is a serious and common condition, but genetic studies to date have been hampered by the small size of the assembled cohorts. In the UK Biobank data set, where RD was ascertained by self-report or hospital records, genetic correlations between RD and high myopia or cataract operation were, respectively, 0.46 (SE = 0.08) and 0.44 (SE = 0.07). These correlations are consistent with known epidemiological associations. Through meta-analysis of genome-wide association studies using UK Biobank RD cases (*N* = 3 977) and two cohorts, each comprising ~1 000 clinically ascertained rhegmatogenous RD patients, we uncovered 11 genome-wide significant association signals. These are near or within *ZC3H11B, BMP3, COL22A1, DLG5, PLCE1, EFEMP2, TYR, FAT3, TRIM29, COL2A1* and *LOXL1.* Replication in the 23andMe data set, where RD is self-reported by participants, firmly establishes six RD risk loci: *FAT3, COL22A1, TYR, BMP3, ZC3H11B* and *PLCE1.* Based on the genetic associations with eye traits described to date, the first two specifically impact risk of a RD, whereas the last four point to shared aetiologies with macular condition, myopia and glaucoma. Fine-mapping prioritized the lead common missense variant (TYR S192Y) as causal variant at the *TYR* locus and a small set of credible causal variants at the *FAT3* locus. The larger study size presented here, enabled by resources linked to health records or self-report, provides novel insights into RD aetiology and underlying pathological pathways.

## Introduction

The UK Biobank is a major international research resource designed to help identify the genetic and non-genetic causes of complex diseases burdening middle and old age (1). Data linkage to the UK National Health Service (NHS) hospital in-patient records, achieved for a large portion of the 500 000 participants, makes it particularly valuable for the investigation of a wide range of conditions. Here, we made use of the UK Biobank to gain genetic insight into retinal detachment (RD), a common condition and cause of emergency ophthalmic intervention.

The processes leading to the vision-threatening separation of the neurosensory retina from the underlying retinal pigment epithelium can be diverse. Break in the retina leading to sub-retinal penetration of vitreous humour characterizes the most frequent RD type, hence called rhegmatogenous RD (RRD). Other forms exist: the far less common tractional form, with no detectable retinal break, and the very rare serous RD, caused by inflammatory or exudative retinal conditions (2).

Annual incidence has been estimated for RRD at 12 per 100 000 population (3) in the UK, peaking in individuals in their sixties (4). Posterior vitreous detachment, a common age-related condition, is thought to be a frequent initiating event (5,6). Trauma, cataract operation, myopia and diabetic retinopathies can precipitate vitreous changes (5) and have all been associated with increased risk of RD. Retinal conditions such as lattice degeneration (7,8) may predispose to retina breaks (holes and tears). Most epidemiological studies have also reported RD incidences to be 1.3- to 1.9-fold higher in men (3,9,10), even when trauma cases are excluded (10,11).

RD has been poorly characterized genetically due to the small sample sizes of collections assembled. A genetic predisposition for RRD has been suggested by family studies, with roughly a doubling of lifetime risk in first-degree relatives of cases compared with that of controls (12,13), and by the, rare, Mendelian syndromic conditions featuring RRD (14,15). The proportion of RRD liability contributed by common genetic variants was estimated at 27% in the very first RRD genome-wide association study (GWAS), which we performed using 867 Scottish cases (16). This GWAS led to the identification of a common coding variant in the CERS2 gene, rs267738, as significantly associated. Several rare mutations in *COL2A1* (in which Stickler syndrome causal variants have been described) have been implicated in familial dominant RRD (17,18). A burden of rare variants in this COL2A1 gene as well as a common intronic variant, rs1635532, have additionally recently been suggested to contribute to idiopathic RRD (19).

Here, we evaluate genetic associations with RD risk, leveraging available information in the UK Biobank. The data linkage to hospitalization records should allow identification of RD cases, with some details. In addition, all UK Biobank participants completed, at recruitment, a touchscreen questionnaire followed by verbal interviews, which included history of RD. This allows capture of potential cases not linked to medical records. This ascertainment is a priori less precise than the hospital records linkage. However, RD, requiring surgical intervention, appears to be a significant ophthalmic event that has a memorable impact on patients (12).

To validate the RD cases ascertained from the UK Biobank, we first assessed self-reported RD using the overlap with hospital admission records where possible, together with the wealth of other information gathered on participants. We then performed a GWAS of RD in the UK Biobank using the union of self-reported and hospital record-linked cases, to maximize power in a sample of RD cases anticipated to be aetiologically heterogeneous. To interpret the genetic associations uncovered, we carried out sensitivity analysis using phenotypic subsets and sought out overlapping associations with high myopia and cataract operation, two well-established conditions increasing RD risks, as well as with clinically ascertained RRD in independent sets from Scotland and England.

Finally, we performed the largest GWAS analysis to date for RD by combining the UK Biobank RD data with the, smaller, studies of clinically ascertained RRD. To replicate our findings, we performed a look-up, in a population-based sample from the personal genetics company 23andMe, Inc. data, for the lead variants of the genome-wide significant signals (study workflow is summarized in Supplementary Material, Fig. S1).

## Results

### Evaluation of UK Biobank self-reported retinal detachment

A total of 1 754 UK Biobank participants self-reported RD (RD-SR) at any one of the three assessment visits. Questionnaire responses were fairly consistent in the baseline visit, with 88% of RD-SR cases also indicating a condition which would prevent them from undergoing spirometry. This compares with 7.7% in all respondents (history of RD is one of the contraindications for spirometry in the UK Biobank protocol). Furthermore, 79.4% of cases reported having had an eye problem other than wearing glasses (compared with 14.7% in all respondents) and 79.8% answering as having had a retinal operation/vitrectomy operation (compared with 0.6% in all UK Biobank participants).

RD-SR cases display significant distributional shifts compared with controls (Table 1), with magnitudes consistent with RD epidemiological reports. They were older (median year of birth 1947 compared with 1951 in controls) and with a 1.3 times greater proportion of men (59.1% compared with 45.1%). Over a quarter had had a cataract operation, in line with prior cataract surgery reported in a fifth to a third of individuals with RD in European cohorts (3,9,20,21). Other self-reported conditions of significant increased prevalence compared with controls include ocular trauma, diabetes, macular degeneration and glaucoma. The distributional shifts are not due to sex bias, as they are maintained in the same gender comparisons (Table 1), for example, for eye trauma, significantly more frequent in men (*P*-value =0.012). When applicable, the median age for first use of lens to correct for myopia was younger, 12-years-old, in cases compared with that in controls, 17-years-old. This self-reported fact has been shown to accurately identify myopia and age at first use to inversely correlate with severity of myopia (22). The shift observed in RD-SR cases towards younger age of onset therefore suggests higher incidence of high myopia, in agreement with it being a well-recognized risk factor for RD (3,11,23).

**Table 1:** Self-reported features in self-reported RD cases and controls in UK Biobank (all ethnicities)

		RD-SR *N* = 1754	RD-SRwithOP *N* = 1391	Controls *N* = 457 255
% Female (ratio female/male)		40.94%^**^ (718/1036 = 0.69)	39.76%^**^ (553/838 = 0.66)	54.84% (250 773/206482 = 1.2)
YOB [med; min-max]	All	1947;1937–1969	1947;1937–1969	1951;1934–1971
	Male	1947;1937–1969	1947;1937–1969	1950;1934–1971
	Female	1947;1937–1968	1948;1938–1968	1951;1936–1970
Cataract *N* (%) % in Female/Male		533 (30.39%)^**^ 29.67%^**^/30.88%^**^	449 (32.28%)^**^ 32.55%^**^/32.10%^**^	6856 (1.50%) 1.62%/1.36%
Cataract op *N* (%) % in Female/Male		456 (26.00%)^**^ 25.49%^**^/26.35%^**^	383 (27.53%)^**^ 27.31%^**^/27.68%^**^	4291 (0.94%) 0.89%/1.00%
Eye trauma *N* (%) % in Female/Male		25 (1.43%)^**^ 0.56%^**^/2.03%^**^	21 (1.51%)^**^ 0.54%^**^/2.15%^**^	553 (0.12%) 0.06%/0.19%
T2D *N* (%) % in Female/Male		24 (1.37%)^**^ 0.97%NS/1.74%^*^	23 (1.65%)^**^ 1.27%^*^/1.91%^**^	3302 (0.72%) 0.51%/0.97%
T1D *N* (%) % in Female/Male		6 (0.34%)^**^ 0.42%^**^/0.29%NS	6 (0.43%)^**^ 0.54%^**^/0.36%^*^	404 (0.09%) 0.08%/0.10%
Diabetic-eye disease *N* (%) % in Female/Male		7 (0.40%) NS 0.14%NS/0.58%NS	6 (0.43%)NS 0.18%NS/0.60%NS	1063 (0.23%) 0.16%/0.32%
Glaucoma *N* (%) % in Female/Male		126 (7.18%)^**^ 6.41%^**^/7.72%^**^	105 (7.55%)^**^ 6.51%^**^/8.23%^**^	4771 (1.04%) 0.91%/1.21%
Macular degeneration *N* (%) % in Female/Male		9 (0.51%)^**^ 0.84%^**^/0.29%^**^	5 (0.36%)^**^ 0.72%^**^/0.12%NS	348 (0.08%) 0.09%/0.06%
Age_at first use of corrective lens for myopia [med; min–max]		12; 1–55	12; 1–50	17; 1–68

RD-SR are UK Biobank participants who self-reported a RD; RD-SRwithOP additionally self-reported to have undergone a retinal operation. Controls are described in Supplementary Note 2. ^**^ and ^*^ denote significant difference compared with control proportion based on a two-sided proportion *z* test at, respectively, 1 and 5% threshold; NS, no significant difference. YOB, year of birth; Med, median; Min, minimum; Max, maximum and Op, operation.

**Figure 1:**
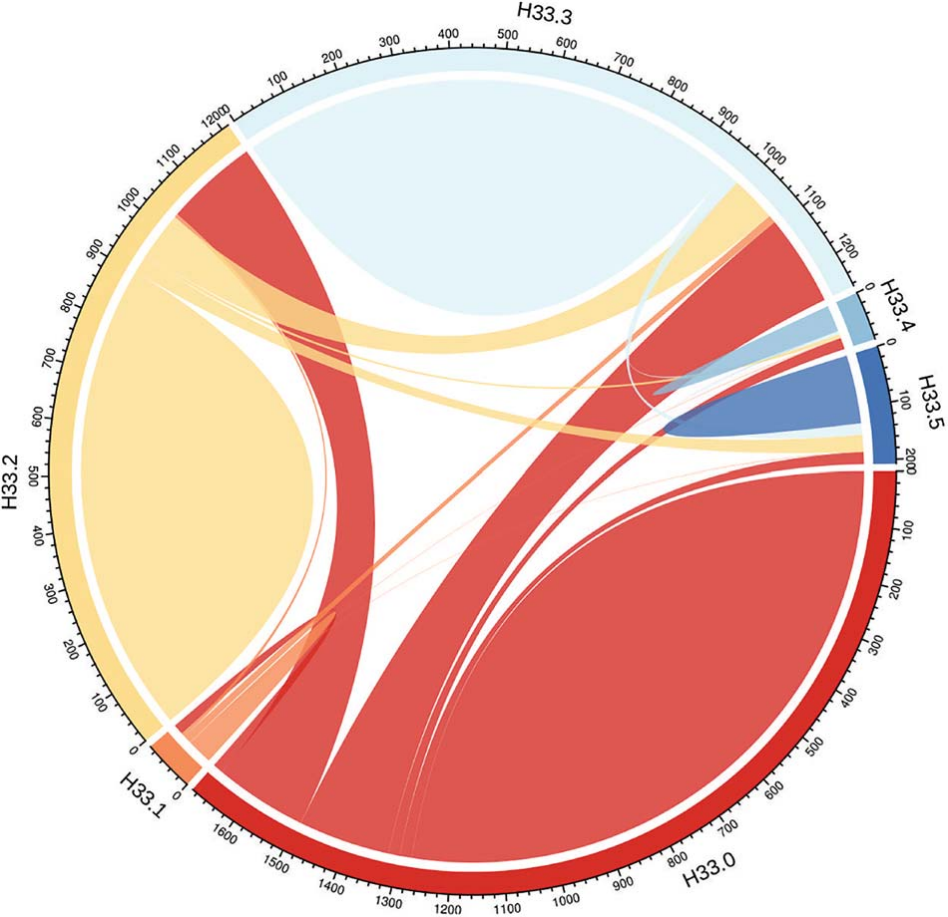
Distribution of ‘RDs and breaks’ ICD10 code H33 subtypes amongst UK Biobank participants. H33 encompasses subclasses H33.0 (RD with retinal break), H33.1 (retinoschisis and retinal cysts), H33.2 (serous RD), H33.3 (retinal breaks without detachment), H33.4 (traction detachment of the retina) and H33.5 (other RDs). The numbers represent the UK Biobank participants (no filtering-all ethnicities included) associated with each code; as only overlap between two codes can be represented, individuals linked to more than two codes are counted several times. The plot was drawn with the R package *circlize* v0.4.3 (73).

Linkage to ‘RD or break’ in hospitalization records was found for 890 (50.7%) of the RD-SR cases (Supplementary Material, Table S1). The highest proportion (~half of those linked to a record, ~25% of total RD-SR cases) corresponded to RRD, ICD10 code H33.0 (RD with retinal break). However, very surprisingly, almost as many of the RD-SR cases (~21%) were linked to the hospital record H33.2, which represents exudative (serous) RD, a very rare form of detachment, suggesting that the ICD subcode entries are likely to be inaccurate. The ~11% of RD-SR cases linked to an H33.3 code (retinal break, no detachment) could also reflect imprecise coding in hospital entries, and/or the breaks are a risk for future RD. Figure 1 displays RD ICD9 and ICD10 entries in the overall UK Biobank participants and illustrates the overlaps between the different subcodes, across hospitalization events.

There seems little gain in restricting RD-SR cases to the individuals who self-report a retinal operation/vitrectomy for greater accuracy: 363 cases would be excluded, but these do not change the overall characteristics of the RD-SR cohort (Table 1 and Supplementary Material, Table S1). Given the enrichment in RD cases that they are likely to represent, we combined RD-SR cases with individuals linked to the ICD10 super code H33 and ICD9 code 361 to increase power in the initial association study (RD-SR-ICD).

### Genetic variants enriched in the UK Biobank SR and ICD-defined retinal detachment cases 

**Figure 2:**
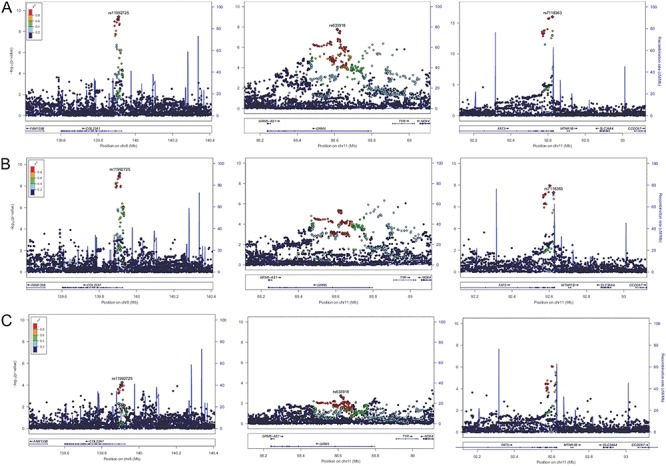
Regional association plots at the three genome-wide significant loci in the primary UK Biobank RD analysis across different case definitions. The *y*-axis represents the negative logarithm (base 10) of association *P*-value and the *x*-axis the position on the chromosome. The lead variants from the RD-SR-ICD discovery study (**A** – *N*_cases_ = 3977) are marked by a purple diamond. The colours of the other variants reflect LD strength (*r*^2^) with the lead variant at each locus. (**B**) Results at the same loci when cases linked to ICD10 codes H33.1 and H33.3 are omitted (*N*_cases_ = 2893). (**C**) Results when cases are restricted to participants with H33.0 code (*N*_cases_ = 1380).

GWAS was carried out in the UK Biobank subset of participants of white-British ancestry. It identified three genome-wide significant signals with common lead variants rs11992725 (MAF = 31.9%, *P*-value = 3.1x10^−10^) on chromosome 8, an intronic variant in *COL22A1*, rs7118363 (MAF = 39.2%, *P*-value = 1.2 x 10^−16^) on chromosome 11, an intronic variant in *FAT3* and rs633918 (MAF = 38%, *P*-value = 1.6 x 10^−8^), also on chromosome 11 and an intronic variant in *GRM5* (Supplementary Material, Fig. S2 and regional plots Fig. 2A).

No genome-wide significant association has so far been published for the *COL22A1* lead variant (or with 1000G proxy variants in high LD, *r*^2^ > 0.6) in the GWAS catalogue or using PhenoScanner (24), as of September 2019. Only recently, the lead variant in *FAT3* has been associated with heel bone mineral density (25) using the UK Biobank data set (*N* = 194 398,*P*-value = 4.65 x 10^−8^). The lead variant in *GRM5* is in LD with variants that have been strongly associated with eye colour (e.g. proxy rs7120151, *r*^2^ = 0.64, *D*′ = 0.94, *P*-value = 2.45 x 10^−10^) (26).

A full list of loci with lead variants displaying an association *P*-value <5 x 10^−6^ is shown in Supplementary Material, Table S2. Two of the 24 suggestive loci (5 x 10^−6^ < *P*-value ≤ 5 x 10^−8^) are amongst the most strongly supported loci associated with common myopia (27): *LAMA2* (identical lead variant) and *BMP3* (reported myopia lead variant rs5022942 (27) in some linkage disequilibrium, *r*^2^ = 0.19, *D*′ = 0.999, with the RD suggestive locus lead variant). Another notable suggestive association is that within the *COL2A1* gene as it is an excellent RD candidate gene (18). The *COL2A1* common variant rs1635532 previously proposed to be associated with RRD (19) displays no association (*P*-value = 0.95) in the UK Biobank RD-SR-ICD, whilst in low linkage disequilibrium with the suggestive lead variant, rs1635554 (*r*^2^ = 0.15, *D*′ = 0.88). The *CERS2* variant rs267738 previously associated with RRD (16) shows nominally significant association, *P*-value = 4.2 10^−3^, with the major T allele increasing risk as previously reported.

The quantile–quantile plot for the GWAS results (Supplementary Material, Fig. S2, *λ*_GC_ = 1.097), and the LDscore regression intercept close to 1 (1.0083, SE = 0.0067) (28), suggest an excess of genetic associations attributable to polygenic effects, compared with the null expectation of no genetic effect. Using a more balanced, 1:3, case/control design, which has been advocated (29) to avoid possible distortion of results for rare variants (which are not analysed here), yields the same top two genome-wide significant signals but with less significant P-values (Supplementary Material, Fig. S3), indicating some loss of power when using a smaller set of control genomes.

Jointly, the common variants tested contribute a heritability on the liability scale, ${h}_{gl}^2$, of 0.23 (*P*-value = 2 x 10^−72^). ${h}_{gl}^2$ calculated using LD score regression was similarly low but significant (0.1378, SE = 0.0194).

### Retinal detachment sub-analyses and genetic overlaps with high myopia and cataract operation in the UK Biobank data set

We carried out sensitivity analyses to tease out phenotypic heterogeneity. Performing the same GWAS analyses after removing cases linked to an ICD code suggesting a break in the retina but no detachment (Fig. 2B, leaving *N*_cases_ = 2 893 for *N*_controls_ = 360 233) did not change the effect sizes of the *COL22A1* and GRM5 lead variants substantially (Supplementary Material, Table S3). The estimated effect for the *FAT3* lead variant, odds ratio (OR) = 1.157, remained significant but fell just outside the 95% confidence interval (CI) of that obtained in the full analysis [1.158, 1.265] (Supplementary Material, Table S3). This suggests that the subset of cases with breaks may contribute more substantially to the *FAT3* signal.

Performing GWAS in the much reduced set comprising only the cases linked to an H33.0 ICD code (*N*_cases_ = 1 380 and *N*_controls_ = 360 233)—which should correspond most closely to RRD cases—does not yield any genome-wide significant associations. However, *FAT3* remains amongst the strongest signals (*P*-value = 8.6 x 10^−7^ for lead variant rs10765567) (Fig. 2C).

In addition to ICD code sensitivity analyses, we looked for evidences in the UK Biobank of shared genetic signals with the two epidemiologically established conditions known to significantly increase RD risk: cataract operation and high myopia. GWAS analyses for high myopia (*N*_cases_ = 2 737 and *N*_control_ = 47 635) and history of cataract operation (*N*_cases_ = 21 679 and *N*_controls_ = 387 283) showed contribution of genetic variants to risk, very strong for high myopia, with LD score regression estimated SNP-based heritabilities, ${h}_{gl}^2,$ of 0.7217 (SE = 0.0642) and very modest 0.0719 (SE = 0.0072) for cataract operation. Manhattan plots for these analyses are shown in Supplementary Material, Figure S4 A–B and the lists of significantly associated loci in Supplementary Material, Table S4–S5. In addition to the top associated locus, intergenic *GOLGA8B*-*GJD2* (lead rs524952, *P*-value = 1.2 x 10^−24^), 9 out of the other 16 high myopia loci show nominal association with RD-SR-ICD (*P*_RD_ < 0.05). All 10 associations show agreement with the epidemiological association (the allele increasing high myopia risk increases risk of RD). Two out of the 20 cataract operation-associated loci, encompassing the eye colour genes *OCA2* and *NPLOC4*, show nominally significant association with RD, with the cataract risk allele increasing risk of RD. The regional high myopia and cataract association plots for the three genome-wide significant RD loci *COL22A1*, *FAT3* and *GRM5* are shown in Supplementary Material, Figure S5 and suggest that those are not driven by a high myopia or cataract association.

Consistent with the epidemiological and phenotypic associations, the overall genetic correlations calculated using LD score regression slopes (30) were significant, 0.46 (SE = 0.08) between RD and high myopia and 0.44 (SE = 0.07) between RD and cataract operation, whilst 0.25 (SE = 0.066) between high myopia and cataract operation.

### Validation of UK Biobank RD genetic associations in data sets with clinically ascertained RRD cases

Genetic variants associated with RD-SR-ICD in the UK Biobank were evaluated for their association with clinically ascertained RRD in two independent data sets. Those were small data sets comprising 980 and 1184 cases, collected in Scottish vitreoretinal surgeries and at the Moorfields London Hospital, respectively. We performed GWAS scans using these clinically ascertained cases and population-matched controls (*N* = 9 706 from Scotland and *N* = 10 000 from London) and genotypes imputed to the same Haplotype Reference Consortium (HRC.r1-1) reference panel as that used for UK Biobank data set (data clean up Supplementary methods 1 and 2).

Association statistics for the lead variants of the three RD-SR-ICD genome-wide significant loci are displayed in Supplementary Material, Table S6. The *FAT3* signal lead variant shows consistent effect direction and magnitude across studies (low heterogeneity index, *I*^2^ = 0, *P*-value = 0.56), with estimated OR for the major allele varying from 1.111 (RRD-London) to 1.186 (RD-SR-ICD). Effect directions at the two other genome-wide significant loci were consistent across all data sets, but heterogeneity in their magnitude was high although not significant (*I*^2^ = 64%, *P*-value = 0.06 for *COL22A1* lead variant, *I*^2^ = 59.7%, *P*-value = 0.084 for *GRM5*’s), indicating OR 95% CI overlap between the three studies.

**Figure 3:**
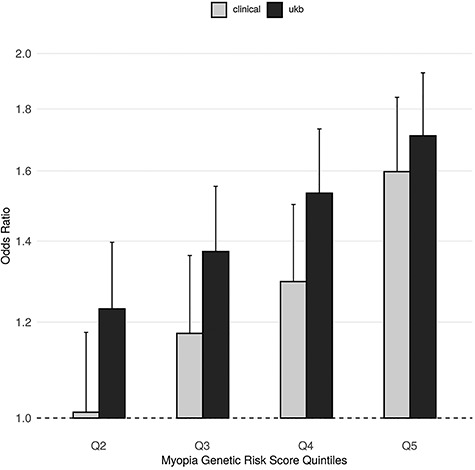
Myopia genetic risk score effect on RD risk using UK Biobank RD-SR-ICD cases or clinically ascertained RRD cases. The effects of consecutive myopia genetic risk score (GRS) quintile on RD (RD-SR-ICD) risk in UK Biobank and on RRD risk in the aggregated clinically ascertained sets are displayed in black and grey bars, respectively. Quintiles were calculated using *N* = 408 972 UK Biobank participants of white-British ancestry. OR for detachment, plotted along the *y*-axis, uses odd in the first quintile as the reference. GRS boundaries for each quintile are given in Figure S6. Error bars denote the OR 95% CI.

Furthermore, using summary statistics from the latest, large-scale, refractive error GWAS (31), we evaluated the effect of a myopia genetic risk score on RD risks in the two types of case ascertainment. The score distributions showed a similar shift towards higher scores for cases in both the UK Biobank study of non-clinically ascertained RD and the data set of clinically ascertained RRD (Supplementary Material, Fig. S6). The myopia genetic score effects on RD risks were of similar magnitudes in both data sets, with significant increase of RD risk, OR_rd_ = 1.711 (CI = [1.518; 1.928], *P*-value = 1.39 x 10^−18^) and OR_rrd_ = 1.598 (CI = [1.387; 1.840], *P*-value = 8.12 x 10^−11^), in individuals in the highest quintile of the myopia genetic risk score relative to individuals in the lowest quintile (Fig. 3). The corresponding OR for high myopia risk in UK Biobank participants was 3.444 (CI = [2.987; 3.970], *P*-value = 4.6 x 10^−65^).

Supported by this range of evidence of common genetic underpinning, a GWAS meta-analysis for all studies (GWAMA) was performed. Of note, the *CERS2* variant rs267738 we previously reported as associated with RRD (16) did not reach nominally significant association with RRD in the analysed London-based data set (which only partially overlaps with the London-based data set used previously (16)), allele T increasing risk, OR = 1.023 (CI[0.925–1.132], *P*-value = 0.65), despite good quality calls for a genotyped variant in high LD with it, rs267734, on the Illumina GSA chip used here.

### GWAMA of UK Biobank and clinical retinal detachment data sets

**Figure 4:**
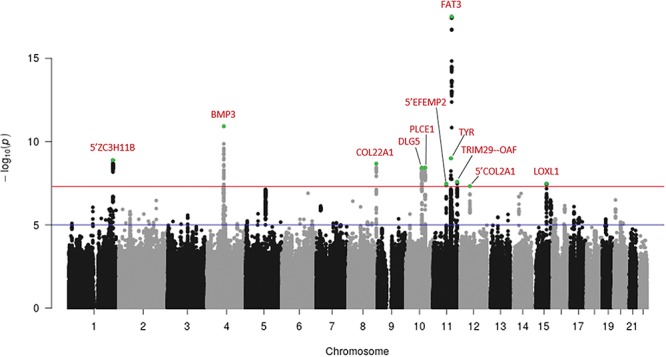
Manhattan plot following meta-analysis of genome-wide association studies using UK Biobank cases (RD-SR-ICD, *N* = 3977) and clinically ascertained RRD cases (*N* = 2164). The *y*-axis represents the negative logarithm (base 10) of the meta-analysis *P*-values and the *x*-axis the location of variants tested. Loci displaying association reaching the genome-wide significant threshold (red line, *P* = 5 x 10^−8^) are indicated in red, with lead variant (lowest *P*-value within a locus) indicated in green. Suggestive level of significance (*P* = 10^−5^) is indicated by a blue line.

Following GWAMA and removal of variants with high heterogeneity across studies, 239 variants distributed across 11 independent loci showed genome-wide significant (*P*-value <5 x 10^−8^) association with RD (Fig. 4; Supplementary Material, Fig. S7 and Table S7 for lead variant regional plots and summary statistics). These loci include *FAT3* and *COL22A1. TYR* is also included, not the physically close *GRM5*, with the common missense variant rs1042602 (TYR S192Y) as lead variant. The A derived allele, strongly associated with light skin colour (32) and absence of freckles (33), increases the risk of RD in all three studies, with high homogeneity (Forest plot Supplementary Material, Fig. S8). In addition, the 5′ *COL2A1* locus, upstream of the known familial RRD causal gene *COL2A1*,as well as *BMP3*, 5′ *ZC3H11B*, *DGL5*, 5′ *EFEMP2*, *PLCE1*, *LOXL1* and an intergenic region between the *TRIM29* and *OAF* genes are also genome-wide significant. The GWAMA had a genomic control inflation factor, *λ*_GC_, of 1.096, and the LD score regression indicates the inflation of type 1 errors to be due mostly (~85%) to true genetic effects (intercept =1.0186 (0.0069), ratio = 0.152(0.056)).

The functional mapping and annotation (FUMA) of genetic association tool (34) was used to annotate the GWAMA results. It called heuristically two independent lead variants at the *BMP3* locus, rs28420618 and rs74764079, based on low level of LD (*r*^2^ < 0.1) between these two physically close genome-wide significant variants. A stepwise model selection, implemented in GCTA (35), indicated association at each of the 11 locus to be captured wholly by the variant with the lowest *P*-value. Further conditioning on each of these lead variants demonstrated that the lead variant effectively tags all of the association signal at each locus, except at *BMP3*. There, conditional analyses suggested two signals, none of which reaching genome-wide significance on its own, tagging each other to some extent, presented by the independent lead variants detected by FUMA (Supplementary Material, Table S8 and Fig. S9). The same two signals underlie genome-wide significant associations at *BMP3* in the recent, large-scale, refractive error GWAMA (31).

Further statistical delineation of credible sets of causal variants was carried out following the method described by Mahajan *et al*. (36) (see methods). Results, shown in Supplementary Material, Table S9, point to one variant with high causal probability (PIP > 0.6), the *TYR* lead variant, rs1042602, and to a small credible set of variants for the *FAT3* signal, encompassing the lead and three linked variants. An alternative variant selection method implemented in FINEMAP (37) was applied to the denser RD-SR-ICD GWAS and resulted in prioritization of very similar sets of variants at *COL22A1* and *FAT3* (Supplementary Material, Table S10).

FUMA annotated a total of 472 candidate causal variants, the 12 independent lead SNPs and all tagged (*r*^2^ > 0.6) variants. Those with distinctive functional annotations are listed in Supplementary Material, Table S11. Two loci harbour variants predicted to have highly deleterious effect (CADD score > 20) (38): the missense and lead variants in *BMP3* (rs74764079) and in *TYR* (rs1042602). High CADD scores (>15) for variants in high LD with lead variant were also notable in *TRIM29*, *COL2A1*, *PLCE1* and *LOXLI*. Evidence of potential transcriptional regulatory attributes could be found at each of the 11 loci (Supplementary Material, Table S11), for example, for every variant of the small credible set established at *FAT3*.

Supplementary Material, Table S12 lists previously reported associations or top associations in UK Biobank PheWAS repositories (PhenoScanner, Global Biobank Engine, or Gene Atlas), for the lead SNPs, all of which except lead at *DGL5* are amongst the top likely causal variants. In addition to the associations to myopia and refractive error already noted, these look-ups highlighted ocular axial length (and height) association for the lead variant in 5′ *ZC3H11B* and retinal macular thickness for the lead, high CADD and highly likely causal *TYR* missense variant.

Gene-based association analysis (Supplementary Material, Table S13) pointed to two additional associated loci, *CMSS1* and *C16orf45 or nearbyRP11-1021 N1.1*. Transcriptional expression in human eye tissues (Supplementary Material, Table S14) shows that many implicated genes, encompassing those defined using the SNP2GENE function in FUMA (*N* = 102, see methods) and the three genes highlighted by the gene-based test, are highly expressed in the retina (neuronal retina or retinal pigmented epithelium). Of note, two GWAS loci have a very limited number of candidate target genes: *COL22A1* is the sole gene at the corresponding locus and *FAT3* together with a gene on another chromosome, *DBI*, implicated by *trans*-eQTL effect of lead variant in a brain expression data set, at the *FAT3* locus. Mouse mutations associated with abnormal retinal morphologies have been described in four genes, located at independent loci: *TYR*, *FAT3*, *C4orf22* and *COL2A1* (Supplementary Material, Table S14).

Gene-set analysis, with the genes prioritized from variants at a permissive GWAS P-value threshold of 10^−5^, shows ‘basement membrane components’ as top enriched GO cellular component pathways (*P*_enrichment_ = 6.9 x 10^−3^) with included genes *EFEMP2*, *COL2A1*, *LOXL1*, *EFEMP1*, COL8A1, *FRAS1*, *LAMA2* and *ACHE*, from independent GWAS loci. The trait-GWAS enrichment (GWAS catalogue e96 2019-05-03) extends overlap with refractive error, myopia, macular thickness and axial length GWAS results (Supplementary Material, Table S15). Five genes (*DUSP12*, *BACS3*, *BMP2, COL8A1* and *PLCE1*), each at independent RD loci, were in addition flagged as overlapping with the glaucoma endophenotype, vertical cup-to-disc ratio GWAS gene set.

### Replication in 23andMe data set

Replication was sought for the 11 variants capturing association signals at the genome-wide significant GWAMA loci by performing association analysis in the large, independent, 23andMe data set. In this data set, the 9 171 RD participants of European ancestry analysed were self-reported according to questionnaire administrated on a web-based interface. Six associations, at *ZC3H11B*, *BMP3*, *COL22A1*, *PLCE1*, *TYR* and *FAT3*, replicate at a Bonferroni corrected significance threshold of 0.0045 (*P*-value = 0.05/11) (Table 2). In all the 11 look-ups, the alleles associated with increased RD risk were the same as in the GWAMA, and all corresponding OR 95% CI overlap or are very close to each other (Table 2). Alternative variants tagging the lead variants at each locus were checked *post hoc*, to account for potential genotyping quality issues, with similar outcomes (Supplementary Material, Table S16).

**Table 2:** Look-up of RD GWAMA associations in the 23andMe data set

					RD GWAMA		RD 23andMe	
SNP	*Locus*	EA	ALT	EAF	OR[CI]	P	OR[CI]	P
rs4373767	***5**′ **ZC3H11B***	T	C	0.63	1.128 [1.086–1.171]	1.30 × 10^−9^	1.045 [1.014–1.077]	**4.03 × 10** ^**−3**^
rs74764079	***BMP3***	T	A	0.97	0.717 [0.642–0.802]	1.20 × 10^−11^	0.825 [0.755–0.901]	**3.02 × 10** ^**−5**^
rs11992725	***COL22A1***	G	A	0.32	1.125 [1.082–1.170]	2.10 × 10^−9^	1.110 [1.076–1.146]	**7.05 × 10** ^**−11**^
rs1248634	*DLG5*	G	A	0.71	0.888 [0.854–0.942]	3.67 × 10^−9^	0.974 [0.942–1.007]	0.122
rs11187838	***PLCE1***	G	A	0.57	0.894 [0.861–0.928]	3.64 × 10^−9^	0.9397 [0.912–0.968]	**4.51 × 10** ^**−5**^
rs7940691	*5′ EFEMP2*	T	C	0.36	1.113 [1.072–1.155]	3.50 × 10^−8^	1.005 [0.974–1.037]	0.766
rs1042602	***TYR***	C	A	0.63	0.888 [0.856–0.922]	1.01 × 10^−9^	0.9499 [0.921–0.9798]	**1.17 × 10** ^**−3**^
rs10765567	***FAT3***	T	A	0.37	0.841 [0.810–0.873]	3.13 × 10^−18^	0.874 [0.847–0.901]	**1.64 × 10** ^**−17**^
rs11217712	*TRIM29–OAF*	T	G	0.31	0.892 [0.857–0.927]	2.65 × 10^−8^	0.970 [0.939–1.002]	6.73 **×** 10^−2^
rs9651980	*5′ COL2A1*	T	C	0.087	1.188 [1.114–1.267]	4.72 × 10^−8^	1.052 [0.997–1.110]	6.01 **×** 10^−2^
rs4243042	*LOXL1*	T	A	0.47	0.889 [0.854–0.925]	3.33 × 10^−8^	0.990 [0.961–1.020]	0.519

EA is the allele on the plus strand for which the effect is reported, ALT is the alternate allele and EAF is the effect allele (EA) frequency in the meta-analysis. Effects obtained from linear mixed models (RD GWAMA) or logistic regression (23andMe) are reported as OR for RD and *P* is the *P*-value. CI, 95% confidence interval. In bold, significant replication.

## Discussion

We explored the use of cases ascertained from a population Biobank for the study of RD. Genotypes from study volunteers with self-reported conditions or linked to medical records are becoming increasingly available to researchers yet, to our knowledge, have never been evaluated for the study of RD.

For a number of conditions, self-report has been shown to be specific while possibly lacking in sensitivity (22,39). Our findings support this to also be the case for RD with the RD self-reported participants in UK Biobank displaying characteristic features well-established for this condition. A more thorough investigation of phenotype accuracy is however warranted, especially with linkage to medical and operation notes increasingly available. With genetic risk effect sizes for common conditions typically small (41), self-reported conditions have the potential to boost power of genome-wide association studies (39,40). For example, the recent large refractive error GWAS (31) exploited the remarkable similarity of genetic findings between analyses of refractive errors derived from a simple online questionnaire and those derived from optical instruments (40).

A major limitation of our work is that while case numbers have been increased by using the UK Biobank data set, we are unable to ascertain which RD subtypes the cases developed. We did flag that the hospitalization records ICD subcodes may be imprecise. The percentage of RD cases subcoded as H33.2, which represents serous RD, was far greater than clinical experience would suggest. Supporting a widespread systematic error with regard to this specific code, seven individuals recruited for the Scottish RRD study were also volunteers for the Scottish population-based GS:SFHS study and all allocated H33.2 in the extracted hospitalization records (two had also the H33.0 code at another hospitalization episode). These seven individuals were definitively phenotyped as RRD by consultant surgeons. This error is likely due to erroneous entries, as further subtypes of H33.0 do exist in the classification of diseases, including H33.02, RD with multiple breaks. However, the linked H33 records have only one digit after the dot. The increased frequency of individuals linked to the H33.3 subtype, corresponding to break in the retina but no detachment, amongst UK Biobank participants who self-reported a RD compared with controls (11% versus 0.14%) could again reflect erroneous coding but is also consistent with a break being a precursor of future RD. The hierarchical structure of diseases in the ICD classifications, exploited by others to improve detection of genetic associations (41), should ensure some degree of specificity. All these considerations justified our combination of all H33 codes for the purpose of our initial analysis, which aimed at identifying genetic factors predisposing to RD risk.

The SNP-captured heritability estimate for RD in the UK Biobank, 23%, appears very similar to that obtained, using the same method, for RRD in the Scottish clinical data set (16). Furthermore, the very identical effects of a genetic load of known myopia risk variants on the UK Biobank RD and clinically validated RRD risks supported that these data sets could be combined to increase risk variant detection. Close to 6  000 RD cases were analysed in the GWAMA, with some very consistent effects across sets. Smaller data sets, such as those analysed so far for clinically validated RD, are prone to false negative as well as ‘winner’s curse’, in which effect sizes reported are upwardly biased (42).

Association with RD was replicated for six genome-wide significant lead SNPs in an independent data set, 23andMe, where definition of cases was entirely self-reported. It is noteworthy that *P*-values in the replication were generally less significant than in the GWAMA, despite a larger number of cases: this could be due to a looser phenotype definition, a web-based setting, some degree of curation of our controls based on ICD codes and self-reported vitreoretinal surgery, younger median age of participants, so that controls could be cases at older age with higher probability or analysis method. The 23andMe analysis used a logistic regression method that may be less powerful in large sample size study than the linear mixed model approach (43).

Many of the GWAMA implicated genes have functions very pertinent to RD aetiologies. There was a clear myopia-related genetic load, in line with expectation. High myopia is the strongest RD increasing risk factor in individuals who have undergone cataract operation, above the type of cataract surgery or history of trauma (11). Myopia is also a prominent feature in ~50% of cases who have never undergone cataract operation (3,44). RD onset under the age of 50-years-old is strongly associated with high myopia (11,20,23). Risk of chorio-retinal abnormalities and posterior RD, often but not always associated with a retinal break, increases with the severity of myopia and greater axial length (45–48). This is a cause for concern, as myopia and, concomitantly, high myopia are showing significant increases in global prevalence (49). Whilst findings of myopia genetic risk factors are not surprising, not all myopia loci may confer increased RD risk, thus informing on myopia, itself a heterogeneous condition. For example, the lead high myopia variant at *PRSS56* (OR = 1.3, CI = [1.218–1.397]) displayed no significant risk for RD. The loci containing genes relating to increased elongation of the eye and basal membrane remodelling are likely to be most relevant to RD. One of the six replicated RD loci, 5′ of *ZC3H11B*, is a newly identified refractive error locus (31) and has been associated with ocular axial length (50) and high myopia (51)*.*

In agreement with a Dutch familial aggregation study (12) suggesting that RRD genetic risk must extend beyond that contributing to myopia risk, at least two of the replicated RD loci, *FAT3* and *COL22A1*, did not appear to exert their effect on RD primarily through myopia nor through cataract operation. Our fine-mapping analysis highlighted a small set of causal variants at the *FAT3* locus, including the lead variant which was also recently associated with heel bone mineral density (25). *FAT3* encodes an atypical cadherin. Its pattern of expression is wide, but its role in the retina is well-established in mice. Mice homozygous for a knockout allele exhibit abnormal neuronal retina, with an additional synaptic layer (52). It is possible that this altered structure would make the retina more prone to breakage, in keeping with the suggestion from the GWAS sensitivity analyses that *FAT3* may be especially concerned with the retinal breaks. *COL22A1* appears to be the only candidate target gene at the corresponding locus and encodes for the collagen, type XXII, alpha 1 chain and a basement membrane collagen with a very restricted pattern of tissue expression (53). It is highly expressed in the retina (mouse and human tissues) where its function is completely unknown. Its role in extracellular matrix adhesion has been advanced in myotendinous junction, following knockdown of COLXXII expression in zebrafish resulting in contraction-induced muscle fibre detachment (54). Remarkably no association has been reported so far for the implicated variants.

The *TYR* and *PLCE1* associations point to retina structure and homeostasis, impacting ocular conditions not limited to RD.

Fine-mapping prioritized the *TYR* association to be driven by the missense variant pS192Y, lead variant in a recent retinal macula thickness GWAS (55). It is likely to act through variation of tyrosinase in the retinal pigmented epithelium (RPE), the single-cell layer of polarized cells that plays a crucial role in maintenance of the neural retina. While a large epidemiological study on prevalence of RRD in albino patients, whose tyrosinase is inactive, is unavailable, pathophysiologies that may concur with RD have been discussed (56). Histological eye sections of mice with an allelic series at the Tyr locus strongly suggest that albino adult mouse retinas are prone to detach compared with wild-type mice (57). Abnormal morphology of the RPE cells and impaired communication between the RPE and neuronal cells, affecting retinal ganglion cell development, have been well documented in albino mouse embryos (58). Compromised gap junctions between RPE and neural retina represent a compelling cause for RD risk.

The *PLCE1* RD lead variant is in LD (*r*^2^ = 0.6, *D*′ = 1) with the reported lead variant for optic nerve cup-to-disc ratio, a glaucoma endophenotype (59), and in complete LD with that recently reported for primary open glaucoma (60). It has not been associated with IOP (61,62). The *PLCE1* gene encodes for the phospholipase C epsilon 1. It is very strongly expressed in the retina, where PLCE1 has been suggested to regulate neuronal intracellular calcium levels and hence impact growth and differentiation (63). Although the glaucoma and RD epidemiological associations could stem from glaucoma developing secondary to a RD operation, a high proportion of glaucoma has been reported, 9.5%, amongst patients undergoing primary operation for RD (64). Our data gives support to the latter’s implications of shared genetic predispositions.

The partitioning of variants based on their effects across traits is a very powerful tool to better understand and separate disease pathological pathways contributing to their aetiologies. For this approach to be thorough and successful, it is clear that well-powered studies are needed and for RD expanding the size of the study will be essential. With our study results largely driven by the availability of self-report and linked hospital records in UK Biobank, future expansion making use of new incident cases over time, the increased health records linkage, and the availability of similar data sets worldwide is a very promising way forward.

## Materials and Methods

### Ethics statement

All cohorts studied were approved by Research Ethics Committees (REC) as detailed below, and all participants gave written informed consent. The UK Biobank study was conducted with the approval of the North-West REC (Reference: 06/MRE08/65). Generation Scotland: Scottish Family Health Study (GS:SFHS) has Research Tissue Bank status from the Tayside Committee on Medical Research Ethics (REC Reference: 15/ES/0040). The REC references for the Scottish RRD study and the Moorfields RRD collection (were respectively MREC-06/MRE00/19 and 10/H0703/97).

### Evaluation of retinal detachment self-report in UK Biobank

The criteria for self-reported RD (SR_RD) cases and controls based on the UK Biobank questionnaires are detailed in S2 Text. Proportions for conditions well documented to be associated with RRD were compared in cases and controls using a two-sided two-proportion *z*-test. The overlap with RD cases (ICD_RD) defined by relevant international classification of disease (ICD) codes from UK NHS hospital patient record transcripts was examined. ICD codes relating to RD were ICD9 code 361 ‘RDs and defects’ and ICD10 codes H33 ‘RDs and breaks’. In total 4 777 (*N* = 3 913 ICD_RD and *N* = 864 SR_RD with no available hospital record of detachment) were identified.

### Data sets for genome-wide association analysis (GWAS)

#### UK Biobank sets

All analyses were restricted to the large subset of participants with genotype data passing quality control and indicative of white-British ancestry (Supplementary Notes).

#### Retinal detachment

In the primary analysis, participants with RD ascertained from self-report or linkage to hospital records were combined (RD-SR_ICD) as cases. Controls were participants who had declared not to have any contraindication for undergoing spirometry (as used in the evaluation of RD self-report), were not cases, and had not self-reported a history of retinal operation/vitrectomy. The discovery GWAS comprised *N* = 3 977 cases and *N* = 360 233 controls; 10 000 controls recruited in the London centres of Barts, Croydon and Hounslow were not included in this analysis and used as independent controls in the analysis of the clinically ascertained RRD cases from the Moorfield collection.

The case-control ratio for the discovery RD analysis was therefore 1:91. GWAS was additionally performed using a more balanced 1:3 case/control ratio for comparison, with controls matched to cases on gender and assessment centre. As effect sizes and *P*-values are influenced by the case/control ratio, a 1:10 ratio was also analysed for the look-up of UK Biobank top results in the clinically ascertained RRD cohorts results and their meta-analysis, so that identical case-control ratios were compared or pooled together.

#### High myopia

N = 2 737 high myopia cases (spherical equivalent refraction (SER) greater than -6 Diopters) and *N* = 47 635 controls (case-control ratio 1:17.4 – Supplementary Notes) were contrasted.

#### Cataract operation

*N* = 21 679 cases and *N* = 387 283 controls (Supplementary Notes, case-control ratio 1:17.9) were analysed.

#### Clinically ascertained RRD cases and population-based controls

##### Scottish set

980 cases from the Scottish RRD study and 9 705 population-matched controls from GS:SFHS not linked to a RD operation (Supplementary Notes, case-control ratio ~1:10) were analysed. 1 136 421 variants with high imputation quality (INFO ≥ 0.8) and MAF > 1% following genotyping analyses detailed in the Supplementary Methods were tested.

##### London set

1 184 cases recruited in London Moorfields eye hospital (Supplementary Notes) and 10 000 gender-matched London centres recruited UK Biobank controls, defined as per UK Biobank analysis of RD, were analysed. In total, 4 727 220 variants with high imputation quality (INFO ≥0.9) and MAF > 1% following genotyping analyses detailed in the Supplementary Methods were tested.

### Genome-wide association analysis

#### Genome-wide association testing

Single-variant GWAS was performed by testing for an additive effect at each reference allele within a linear mixed model to account for population relatedness and geographic structure. All analyses apart from one were performed using BOLT_LMM v1.3 (43,65) which implements fast parallelized algorithms to analyse large (*N* > 5 000) data sets. The London RRD set analysis was run using GCTA (35) as low heritability estimation prevented BOLT_LMM v1.3 from carrying out the genome scan. Polygenic contribution to trait can be modelled using a mixture of Gaussian priors on SNP effect sizes (default model allowing uneven effect sizes) or a single-Gaussian prior (standard infinitesimal model) in BOLT_LMM v1.3. Reported association p-values are those obtained for the default BOLT_LMM model, but all were identical or very close to those obtained under the standard infinitesimal model in our analyses. Covariates in the models were age (coded as five consecutive year of birth bins) and sex and, in addition, for the UK Biobank data set, recruitment centre, genotyping array, genotyping batch and the 10 first components of the centrally performed UK Biobank principal components analysis. Only common to low frequency, MAF > 1%, variants were analysed given the small size of the case samples and the risk of false-positive associations for rarer variants in the unbalanced case-control samples. In the UK Biobank data set, recent simulations from the BOLT-LMM developers (43) show no inflation of type 1 error, for a significance threshold *α* of 5 x 10^−8^, at this MAF threshold with case fraction of 1% or higher.

Individual RD studies (each using a 1:10 case-control ratio to harmonize effect sizes) summary statistics were meta-analysed using the inverse variance fixed effect scheme implemented in the software METAL (66). Variants with Cochran’s *Q*-test heterogeneity of effect greater than 75% were filtered out.

Effect sizes on the observed scale were converted to log(OR) following the formula described by Pirinen *et al*. (67).

## Replication

Replication analysis of 11 loci was performed using self-reported data from a GWAS including 9 171 cases and 406 168 controls of European ancestry, filtered to remove close relatives, from the 23andMe, Inc., customer database. All individuals included in the analyses provided informed consent and answered surveys online according to the 23andMe human subject protocol, which was reviewed and approved by Ethical & Independent Review Services, a private institutional review board (http://www.eandireview.com). Cases were defined as those who reported either retinal tears or RD; controls were defined as those who reported not having either retinal tears or RD.

All 11 variants assessed for replication were imputed in this study and were amongst the 21 747 472 variants passing quality control in the GWAS. Association test results were computed using logistic regression assuming additive allelic effects, including age, sex, the top five principal components of ancestry and genotyping platform as covariates in the model.

## GWAS evaluation

Manhattan plots were drawn using the qqman library in R and QQ plots using a local function. Regional association plots were plotted using LocusZoom: http://locuszoom.sph.umich.edu/. Inflation of type 1 error not accounted for by polygenicity was estimated using the LD score regression intercept (28) computed by the LDSC software. Z-scores calculated from the GWAS summary statistics from genotyped and well-imputed (INFO ≥ 0.8) variants were regressed on precalculated LD scores (of European ancestry from the 1000G phase 1 reference panel). For one GWAS, the Scottish RRD set, the ratio $\left(\mathrm{intercept}-1\right)/\left(\mathrm{mean}\left({\chi}^2\right)-1\right)$, which measures the *proportion* of the inflation in the mean ${\chi}^2$ that the LD Score regression intercept ascribes to causes other than polygenic heritability, was greater than 20%, and the LD score regression intercept was used as inflation factor to apply genomic control on the results (68).

Manually, a locus was defined as a region with significant variants within a 500kb region centred on the lead variant (that of the lowest *P*-value in the region) and contiguous loci merged. Adjacent ‘loci’ with variants displaying LD measure *r*^2^ > 0.1 across loci, e.g. HLA region for cataract operation GWAS, were merged into one locus. GWAMA loci were defined and annotated within FUMA with the default clumping procedure (first clumping parameter *r*^2^ = 0.6, second *r*^2^ = 0.1) unless for fine-mapping investigation (*r*^2^ = 0.1 for both clumping) to define larger regions.

## Independent signal detection and fine-mapping

The loci were investigated using GCTA v1.91.4beta for stepwise variable selection and conditional and joint (COJO) analyses using a subset of data including ~ 10 000 white-British UK Biobank participants as reference for linkage disequilibrium patterns. GWAMA results, or the conditional analysis results for the region identified with more than one independent lead, were used to calculate approximate Bayes factor (abf) using Wakefield’s formula (69), as implemented in the R package gtx v.2.0.1. Following Mahajan *et al*. (36), the posterior probability for a variant to drive association (PPA) was then calculated as the abf of the variant divided by the sum of the abfs in the region. The 99% credible sets of a region were derived by summing the posterior probabilities in descending order until the cumulative posterior probability was >99%.

Fine-mapping was also performed, on the three loci identified in the discovery GWAS, using FINEMAP v1.3.1 (37). This software uses shotgun stochastic search algorithm to identify the most likely causal configurations of the region. Each variant within a found credible set is defined by its posterior inclusion probabilities (PIP) of being causal and its log_10_BF (Bayes factor) which quantifies the strength of causality evidence.

## Myopia genetic risk score

A myopia genetic risk score (GRS) was derived from 71 out of 140 genome-wide significant lead variants for refractive error (*P* ≤ 5 x 10^−8^) from a large-scale meta-analysis of studies (CREAM and 23andMe meta-analysis) (31) independent from our discovery studies. The 71 variants passed QC in our three investigated cohorts and are listed in Supplementary Material, Table S17. The GRS was constructed as the sum of the additive imputed dosage of the alleles increasing myopic refractive error, weighted by their effect on refractive error. Effect on RD risk was assessed using logistic regression with the following covariates: year of birth bin, sex, assessment centre, batch, genotyping array and five first PCs. The analysis using UK Biobank participants was restricted to those unrelated within the white-British ancestry subset (pairwise kinship coefficients lower than 0.0313).

## Functional annotation and gene mapping

Functional annotation was performed using FUMA (34) v1.3.5, a recently developed integrative tool leveraging information from many biological data repositories. FUMA heuristically defines independent lead SNPS within loci if they are in low LD *r*^2^ < 0.1 (here using the UK Biobank white British set as reference panel). These variants, as well as those, tested or not, in high LD (*r*^2^ ≥ 0.6) with them in the reference panel, were annotated for predicted protein-altering or regulatory features following annotations from ANNOVAR, 2017-07-17 update (http://annovar.openbioinformatics.org), RegulomeDB (http://www.regulomedb.org/), eQTL repositories (with note that none is available as yet for eye tissues) and the ChromHMM predicted 15 core chromatin states derived from ENCODE and ROADMAP repositories. SNP-trait known associations from the GWAS catalogue (e96_r2019-05-03) were also part of FUMA annotations. The SNP2GENE function in FUMA identified putative target genes in an exhaustive manner: by physical mapping to the loci physical locations which are defined liberally by variant coordinates in high LD (>0.6) with any lead and ‘independent significant’ variants which display medium LD with the lead variants (r2 between 0.1 and 0.6) or based on genes whose expression has been significantly associated with those GWAS variants. eQTL repositories used include GTEx v6 and v7, BIOS QTL browser and BrainSpan, amongst others.

## Gene-based analysis and gene-set analysis

These analyses were performed using MAGMA v1.07 (70) within FUMA. Gene-based P-values are derived from an approximation of the sampling distribution of the mean of the χ^2^ statistic for the SNPs in a gene, with gene coordinates that from NCBI 37.3 and LD pattern to account for dependency of SNP *P*-values that of the UK Biobank white British reference data as input parameters. Genome-wide threshold of significance for the gene-based *P*-values was set to 2.68 x 10^−6^, following Bonferroni correction for the *N* = 18 625 genes evaluated.

Gene-based *P*-values converted to *Z* values and a between-gene correlation matrix are used as input to perform gene-set enrichment tests (70). These tests are based on expectation of a hypergeometric distribution for the null hypothesis of no enrichment. The raw *P*-values are adjusted following the Benjamini and Hochberg procedure (FDR set at 5%) to account for multiple testing within each gene-set category. Predefined gene sets from the molecular signature database MsigDB v6.1 (http://software.broadinstitute.org/gsea/msigdb) were used.

## Trait heritability and genetic correlations

The heritability attributable to the joint contribution of genetic variants tagged by the common variants tested, ${h}_g^2$, can be calculated using the variance component estimates from the models fitted to perform the GWAS. It was converted to a case-control ratio-independent heritability, on the scale of liability, ${h}_{gl}^2$, using the formula (1) discussed in (71) or (2) when controls were also selected using a trait threshold (72) for the analysis of high myopia:

(1) \begin{equation*} {h}_{gl}^2={h}_g^2\times \frac{K\left(1-K\right)}{z^2}\times \frac{K\left(1-K\right)}{P\left(1-P\right)} \end{equation*}

(2) \begin{equation*} {h}_{gl}^2=\frac{h_g^2}{P\left(1-P\right){\left(\dfrac{z_U}{K_U}+\dfrac{z_L}{K_L}\right)}^2} \end{equation*}

where *P* is the prevalence of the binary trait in the analysed sample (e.g. for the largest RD analysis in UK Biobank: 3977/(360 233 + 3977) = 1.1%) and *K* is the prevalence in the population of matching age group as that of the UK Biobank population. We made the assumption that conditions’ prevalences in the UK Biobank sample are similar to that of the UK population; for RD, assuming *K* = *P* was close to a previous UK RRD estimate of incidence of 12 per 100 000 population-year (4) with median age of 59-year-old in the sample. For high myopia, cases corresponded to the 3.76% upper tail of the refractive error distribution and controls to the 65.5% lower tail. *z*^2^ is the squared ordinate of a standard normal curve at the *K* quantile.

${h}_g^2$ and ${h}_{gl}^2$ were also estimated using the linkage disequilibrium (LD) score regression method (28). Genetic correlations (*r_g_*) were those obtained by regressing the product of test statistics against LD score (30) as implemented in the LDSC software.

## GWAS look-up

Four GWAS results repositories were used: the GWAS catalogue (e96_r2019-05-03), PhenoScanner (as of September 2019), the Global Biobank Engine, Stanford, CA (URL: http://gbe.stanford.edu) (GBE) [March 2018] and the GeneATLAS, University of Edinburgh (URL: http://geneatlas.roslin.ed.ac.uk/) [March 2018]. In the Global Biobank Engine, over 2 000 UK Biobank traits (including RD defined as in our discovery analysis) have been analysed in a first broad brush pass, on 337 000 unrelated participants of white British ancestry. The analysed traits include traits measured in smaller subsets of individuals in recall assessments, including the ophthalmologic assessment (e.g. refractive error as a quantitative trait is analysed for *N* = 82 752 participants). The GeneATLAS shows GWAS results for 778 traits measured in the complete set of UK Biobank participants of white British ancestry, using both related and unrelated individuals; results for the genotyped variants can be queried using the web interface.

## Supplementary Material

SupplementaryTables_R2_ddz294

SupplementaryData_R2_FORSUBMISSION_ddz294

## Data Availability

Summary GWAS statistics for the RD GWAMA presented here are available from the University of Edinburgh Digital Repository DataShare (https://datashare.is.ed.ac.uk/handle/10283/3511); summary GWAS statistics for individual UK Biobank trait (high myopia, cataract operation and RD-SR-ICD) are to be returned to the UK Biobank.

## Web Resources

UK Biobank Access Management System, http://www.ukbiobank.ac.uk/register-apply/; HRC variants, http://www.haplotype-reference-consortium.org/site; LocusZoom: http://locuszoom.sph.umich.edu/; NHGRI-EBI catalogue of published genome-wide association studies, http://www.ebi.ac.uk/gwas/; Phenoscanner, http://www.phenoscanner.medschl.cam.ac.uk/phenoscanner; LDSC, https://github.com/bulik/ldsc; BOLT, https://data.broadinstitute.org/alkesgroup/BOLT-LMM/; FUMA, http://fuma.ctglab.nl/; GCTA, https://cnsgenomics.com/software/gcta/; gtx, https://github.com/tobyjohnson/gtx; Haploreg, http://archive.broadinstitute.org/mammals/haploreg/haploreg.php; LD calculation tool, https://www.ensembl.org/Homo_sapiens/Tools/LD/; CADD pre-calculated scores, http://cadd.gs.washington.edu/ and GTEx portal, https://gtexportal.org/home/
